# Supplementary material for: GO-TiO2 as a Highly Performant Photocatalyst Maximized by Proper Parameters Selection
Source: Int J Environ Res Public Health. 2022 Sep 20;19(19):11874. doi: 10.3390/ijerph191911874 (PMC9565788; doi:10.3390/ijerph191911874)
Supplement: Supplementary file 1 [file ijerph-19-11874-s001.zip › ijerph-1835878-supplementary.pdf]

SUPPLEMENTARY MATERIAL

GO-TiO_2 as a well-performant photocatalyst maximized by proper parameters selection

Aida M. Díez[1,2,*], , Yury V. Kolen´ko[1], Marta Pazos[2] and M. Ángeles Sanromán[2]

[1] Nanochemistry research group, International Iberian Nanotechnology Laboratory, Avenida Mestre José Veiga s/n, 4715-330, Braga, Portugal

[2] *CINTECX, Universidade de Vigo, Grupo de Bioingeniería y Procesos Sostenibles, Departamento de Ingeniería Química, Campus Lagoas-Marcosende, 36310* Vigo, Spain.

*adiez@uvigo.es

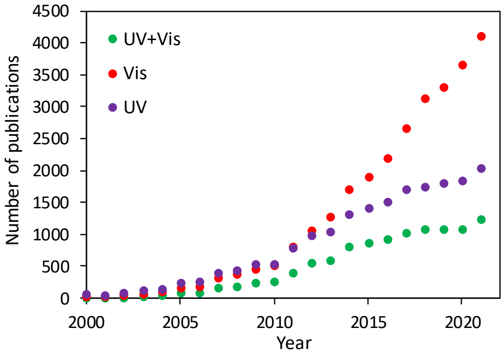

Figure S1-SM. Number of publications indexed on Scopus by searching for photocatalysis+ UV, photocatalysis + Visible or photocatalysis+ Visible and UV.

Table S1-SM. Characteristics of the real working matrixes on the study.

**Parameter (units)**	**Physical treated water**	**Physical+biological treated water**
pH	7.5	7.2
Conductivity (mS/cm)	1.135	1.006
Chemical Oxygen Demand (mg O_2/L)	247	46
Suspended solids (mg/L)	66	19

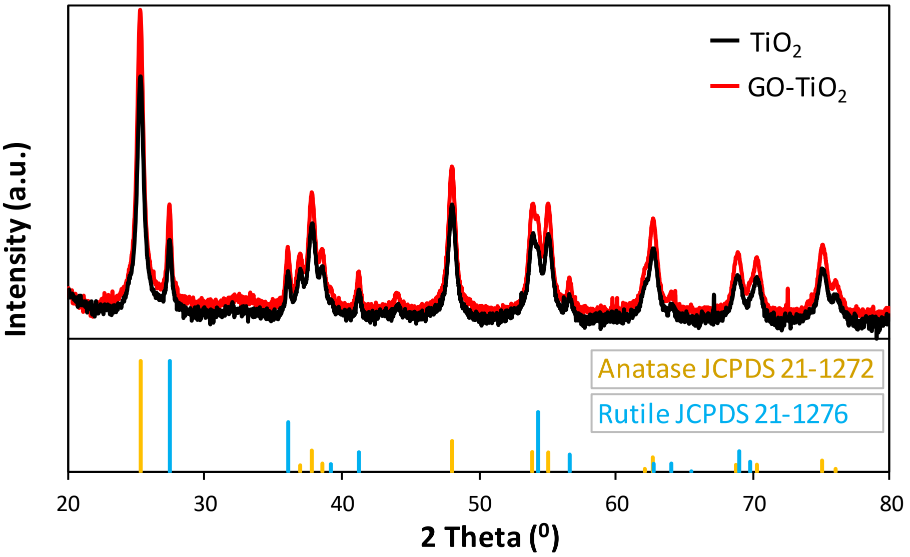

Figure S2-SM. XRD spectra of TiO_2 (black) and GO-TiO_2 (red) catalysts.

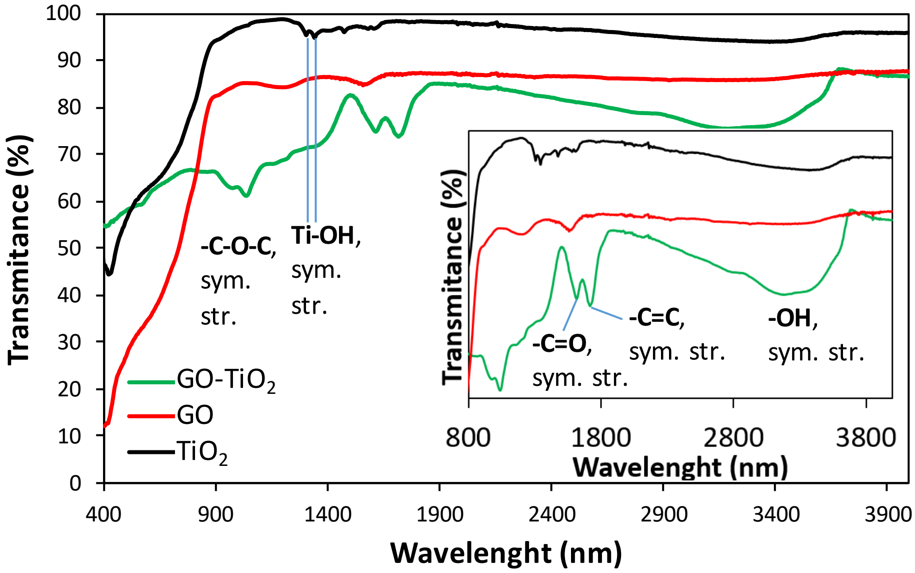

Figure S3-SM. FTIR spectra of TiO_2 (black), GO (green) and GO-TiO_2 (red) compounds. The insert figure is the zoom in of the spectra between 800 and 3700 nm. Sym. str.=symmetric stretching.

Table S2-SM. Porous characteristics of TiO_2 and GO-TiO_2.

Photocatalyst	Surface area (m^2/g)	Pore volume (cm^3/g)	Pore diameter (nm)
**TiO_2**	61.265	0.519	34.3
**GO-TiO_2**	73.197	0.742	39.9

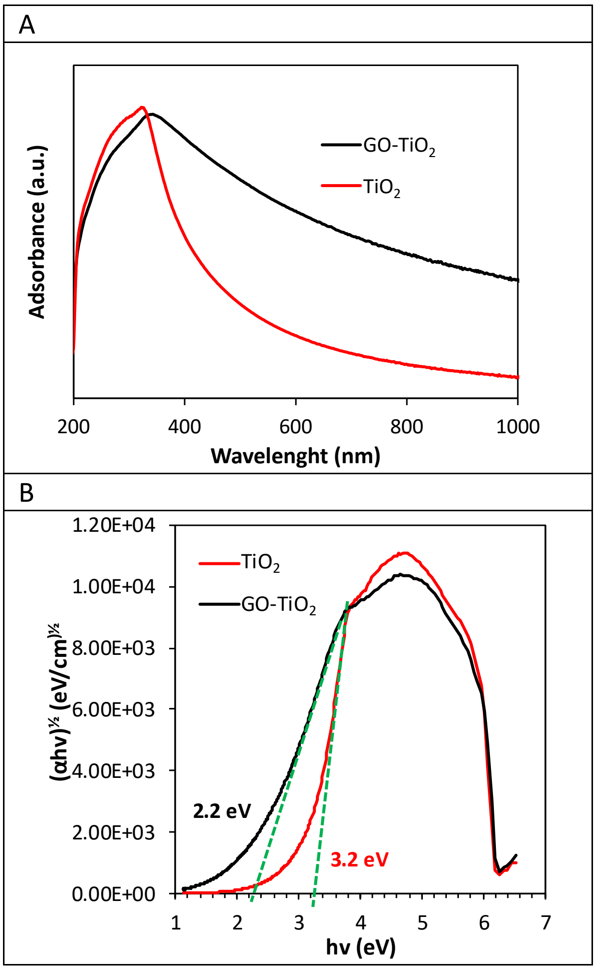

Figure S4-SM. A: UV-Vis spectra of TiO_2 and GO-TiO_2.B. Tauc plots of both TiO_2 and GO-TiO_2 photocatalysts.

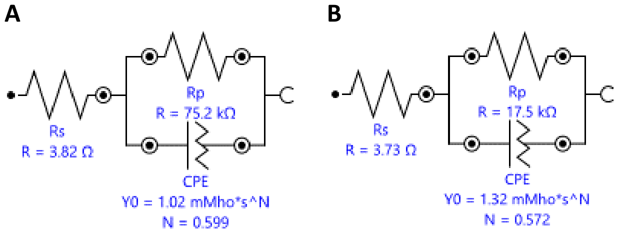

Figure S5-SM. Fitted reactors to the Nyquist results for TiO_2 (a) and GO-TiO_2 (B) catalysts.
